# The importance of providing sterile wound culture before wound closure in Fournier's gangrene

**DOI:** 10.1016/j.ijscr.2025.110845

**Published:** 2025-01-06

**Authors:** Akif Erbin, Çagri Sevik, Direnc Ozboru, Halil Lutfi Canat

**Affiliations:** Department of Urology, Basaksehir Çam Sakura City Hospital, Istanbul, Turkey

Dear Editor,

We read with great interest the study titled “Fournier gangrene in elderly men: Two case reports” [1]. The recent report by Chhetri et al. on two cases of scrotal Fournier's gangrene in elderly males highlights the complexities involved in managing this life-threatening condition, stressing the importance of timely intervention and a multidisciplinary approach. In this context, they stated that following surgical debridement, the essential phase of local wound management involves techniques such as vacuum-assisted closure devices and wet-dry dressings, and patients typically necessitate reconstructive surgery with sufficient tissue coverage for the affected region after granulation tissue has formed. In the study, standard tissue cultures were obtained before the operation in both patients, adequate empirical antibiotics were administered, and following the treatments, the first patient received skin grafting while the second patient got primary wound repair without sterile wound culture control. The crucial question at this juncture is whether the providing of sterile wound cultures prior to wound closure, especially in cases requiring grafts or flaps, is necessary or not.

Empirical and culture-directed antibiotic therapy should persist until the infection is controlled. Initial and subsequent wound cultures are essential in identifying bacteria and customizing antibiotic treatment during the active infection phase. However, cultures can sometimes yield colonizing or contaminating organisms, which might not indicate active infection. Specific guidelines or studies recommending sterile wound cultures immediately before wound closure are limited [2]. A sterile wound culture prior to closure is not a universal requirement but may be pursued in specific cases if there is doubt about infection resolution. The achievement of tissue culture sterility after the antimicrobial treatment can markedly improve the success rates of reconstructive procedures. However, the problem here is that prolonging closure unnecessarily to achieve a sterile wound culture may delay recovery without added benefit.

In conclusion, the decision to close the wound is based on culture results, not solely on clinical judgment and the appearance of the wound, especially in risky and complicated patients where the condition of the wound is suspicious. The first case, in particular, is highly complex and was repaired with a graft. A sterile wound culture could be seen in this case. We think that providing a sterile wound culture might be beneficial in terms of graft infection, particularly in complex cases involving graft repair.

## Author contribution

*Study concept and design*: Akif Erbin, Cagri Sevik, Direnc Ozboru, Halil Lutfi Canat.

*Data analysis*: Akif Erbin, Cagri Sevik, Direnc Ozboru, Halil Lutfi Canat.

*Drafting of manuscript*: Akif Erbin, Cagri Sevik, Direnc Ozboru, Halil Lutfi Canat.

*Critical revision of the manuscript*: Akif Erbin, Cagri Sevik, Direnc Ozboru, Halil Lutfi Canat.

## Consent

The study does not involve any human or animal participants.

## Ethical approval

Since the presented study was a letter to editor study, no ethics committee approval was required.

## Guarantor

The study is a letter to editor study.

## Research registration number

Not applicable.

## Funding

The author(s) received no financial support for this letter to editor study.

## Conflict of interest statement

We have no conflicts of interest to disclose.
